# Healthcare Professionals' Attitudes and Practice of Sexual Health Care: Preliminary Study for Developing Training Program

**DOI:** 10.3389/fpubh.2020.559851

**Published:** 2020-10-16

**Authors:** Sung-Hee Ahn, Jung-Hee Kim

**Affiliations:** College of Nursing, The Catholic University of Korea, Seoul, South Korea

**Keywords:** sexual health, healthcare professionals, practices, attitude, educational needs

## Abstract

Health professionals contribute toward addressing the sexual health care (SHC) of cancer patients, given the frequency of contact with their patients. This study investigated nurses' and physicians' SHC attitudes, practices, and educational needs relating to cancer patients' SHC. Using a cross-sectional study design, we assessed South Korean health professionals' attitudes, practice, and training needs related to cancer patients' SHC. Differences in attitudes and practices among the groups were analyzed *via* an independent-samples *t*-test, ANOVA, and ANCOVA using SPSS. The demographic characteristics, including sex, marital status, and age, were associated with SHC attitudes and practices. There was a significant difference in the level of nurses' and physicians' attitudes regarding SHC. Compared to nurses, physicians were found to hold a more positive attitude toward SHC. Nurses performed practices pertaining to reproductive care significantly more than physicians after controlling for demographic variables. A small correlation was found between scores on the SHCS–A and the SHCS–P among the groups. Participants' educational needs regarding SHC included changes in sexual function, safe sex during treatment, reproductive health, and sexual counseling approaches. Equipping oncology nurses and physicians with the knowledge to extend their roles by managing cancer patients' sexual function, psychological and social problems, and reproductive care would be effective. Additionally, oncology nurses who are mainly women and relatively younger than physicians ought to enhance their skills in communicating with and counseling male cancer patients.

## Introduction

Sexual health, together with quality of life, has increasingly been recognized as a vital component of cancer care (1, 2). Regardless of age, relationship status, and disease type and its treatment, most cancer patients may experience sexual dysfunction (3). Sexual dysfunction is common among most patients across cancer types; it includes problems with sexual behavior, pleasure, intimacy, and reproduction (4, 5). Moreover, chemotherapy, surgery, radiation therapy, and hormone therapy have negative effects on sexual function and patients' organs (4, 6). Erection problems, ejaculation dysfunction, and loss of sexual desire are reported to be 70–100% among male cancer survivors during treatment (3, 7). Thirty to seventy percent of female cancer survivors experience vaginal dryness, dyspareunia, premature menopause, and loss of sexual desire (4, 7). Changes in body image and psychological stress could also contribute to sexual dysfunction (5, 7).

Although cancer patients reported educational needs relating to sexual health after a cancer diagnosis (1), only 37% of patients indicated having received information regarding an altered body image and sexual dysfunction after surgery (8). Healthcare professionals must be educated in the area of sexuality and emphasize sexual health as an integral aspect of well-being (9). Due to their frequent contact with cancer patients, oncology nurses and physicians can play an essential role in addressing patients' sexual health care (SHC) needs (3, 10).

Despite the widespread recognition that SHC is a crucial element in the quality of life of cancer survivors, several barriers to its provision by healthcare professionals have been identified (10–12). For example, the discussion of sexual functioning is still repressed in the patient–nurse relationship, and 46–62% of nurses are not aware of cancer patients' sexual problems (13, 14). Nurses and physicians must also be aware of their attitudes and practices regarding sexual health (15, 16). Differences in attitudes toward addressing sexual health have been observed between healthcare professionals (17). Over 80% of oncology nurses and physicians have agreed that they have a responsibility to discuss sexual health with their patients (18, 19), whereas surgical oncologists cited lack of time (39.9%) and patients' older age (50.6%) as common reasons for avoiding such discussions (18), and 63% of oncology nurses reported lack of training and knowledge in this regard (19). It is unclear as to whether the groups are directly comparable, as previous studies did not use similar measures to assess attitudes toward SHC.

Healthcare professionals' attitudes and practices relating to SHC also differ according to general characteristics or personal attributes (15, 18, 19). For example, older and male surgeons are significantly more likely to discuss patients' sexual concerns (19). Nurses with more oncology experience, more sexual health knowledge, and higher academic qualifications have reported feeling more comfortable discussing cancer patients' sexual concerns (19). To address the gaps between their demanding roles and everyday practices, and patients' needs and concerns, the evaluation of nurses' and physicians' attitudes and practices is therefore critical, considering variations in these general characteristics (1).

Limited attention has been given to SHC training for cancer patients in the healthcare setting. Effective training will enable healthcare professionals to develop the knowledge, attitudes, and practices required for clinical practice in SHC (20). Skelton and Matthews (21) developed an education program on sexual history based on healthcare professionals' perspectives. Similarly, training for rehabilitation teams in SHC was developed and evaluated among SHC health professionals (20). However, no such program has focused on dealing with various sexual problems in cancer patients. In this study, we investigated nurses' and physicians' SHC attitudes, practices, and training needs relating to cancer patients' SHC to develop a SHC training program for health professionals.

## Methods

A cross-sectional survey design using convenience sampling was employed to assess oncology nurses' and physicians' attitudes, practices, and educational needs relating to cancer patients' SHC.

### Data Collection

This study was performed at two tertiary hospitals in South Korea. This study was reviewed and approved by the Ethics Review Board of D University in Korea. Using a two-tailed significance level of 0.05 and a statistical power of 80%, a sample of 34 physicians and 68 nurses, with a 1:2 ratio, was required to detect a medium effect size (14).

With the permission of the one of the hospital administrators, a total of 70 nurses and 40 physicians at the participating hospital were recruited from a university in Cheonan. Only full-time registered nurses or physicians who had cared for cancer patients within the last 6 months were eligible to participate in the study. Trained research assistants recruited eligible participants. After informing prospective participants of the study aim, the research assistants provided verbally consenting prospective participants with a sealed envelope consisting of the self-completed questionnaire and assurance of anonymity and confidentiality. Data collection using self-completed questionnaires was conducted during day and evening shifts to facilitate the inclusion of nurses in all shifts. One hundred questionnaires from 70 nurses (response rate: 100%) and 30 physicians (response rate: 75%) were returned.

### Measurements

The Sexual Health Care Scale–Attitude (SHCS–A) was used to measure participants' attitudes toward SHC. This scale has satisfactory construct and concurrent validity (15). The instrument's subscales are discomfort with providing SHC information, feeling uncertain about patients' acceptance of SHC, being afraid of colleagues' negative responses, and lack of environmental support (15). Some of the items on this scale were “I am afraid patients would feel that their privacy was invaded if I asked specific questions about sex,” “I am afraid my colleagues would consider it unusual for me to deal with patients' sexual issues,” and “I am afraid patients would feel that their privacy was invaded if I asked specific questions about sex.” The scale comprises 17 items measured on a three-point scale ranging from 1 (*agree*) to 3 (*disagree*). The total score ranges from 17 to 51, with higher scores indicating a more positive attitude toward SHC. The Cronbach's alpha for this scale was 0.922 in this study.

The Sexual Health Care Scale–Practice (SHCS–P) was also used in this study. The SHCS–P consists of 21 items, each with a possible score of either 1 or 0, with a total score ranging from 0 to 21 (14). Higher scores indicate more frequent provision of SHC. Some of the items on this scale were “counseling and education regarding a decrease in sexual desire” and “assessment of changes in body image.” The instrument's four subscales are “practice in relation to sexual function,” “psychological factors,” “social problems and records,” and “reproductive care.” The Kuder-Richardson Formula (KR-20) for this scale was 0.869. KR-20 is a special type of Cronbach's alpha, computed for dichotomous scores. Standard references for item analysis consider a KR-20 > 0.70 to indicate good reliability (20).

As the SHCS–A and SHCS–P were originally developed for oncology nurses, a surgical physician at a university hospital who had research experience in sexual health and instrument development reviewed the instruments' appropriateness for the target population, particularly physicians. Since all items reflected the variables being measured, no modifications were made to the instruments.

The questionnaire included four questions on participants' demographic information, including gender, marital status, age, and religion, as well as two questions regarding the healthcare profession and participants' area of interest or occupation. The presence of educational needs and responsibility for the SHC of cancer patients were assessed through “Yes,” “No,” or “Unsure” responses to the question, “Do you need education in SHC for cancer patients?,” “Do you assume responsibility for the SHC of cancer patients?,” and “Have you ever received training in SHC?”

Moreover, the educational needs of the nurses and physicians were described comprehensively in response to open-ended questions. These were “What should health professionals know about SHC?” and “What are your educational needs for practicing SHC on cancer patients?” Answers were sorted and compiled into response categories and subcategories.

### Analysis

The chi-square test was used to ascertain the group differences of the demographic characteristics between nurses and physicians. The normality of data was confirmed using Kolmogorov–Smirnov and Shapiro–Wilks tests. Differences in the sum score in attitudes and practices regarding SHC based on demographic characteristics were divided based on sex, marriage status, and age and were analyzed through an independent-samples *t*-test and ANOVA. To test for differences of the subdomain scores of attitudes and practices regarding SHC among nurses and physicians, independent-samples *t*-test and ANCOVA after controlling the demographic characteristics were utilized. Pearson's correlation coefficients were used to determine the correlation between attitudes and practices in each nurse and physician group and the whole group (Version 21.0; SPSS Inc.). Also, qualitative data from the participants' narratives were analyzed thematically.

## Results

### Participants' Characteristics

The nurses' and physicians' mean (*SD*) ages were 31.44 years (*SD* = 7.14 years) and 42.93 years (*SD* = 4.14 years), respectively. All the nurses were female, 47 (67.1%) of whom were unmarried. Twenty-six (89.7%) of the physicians were male, and all of them were married. Compared to physicians, nurses were relatively younger (χ^2^ = −10.07, *p* < 0.001), predominantly female (χ^2^ = −81.98, *p* < 0.001), and unmarried (χ^2^ = 38.00, *p* < 0.001).

Twenty-seven participants (27%) reported their area of interest or occupation to be internal medicine; 32%, surgery; 38%, obstetrics and gynecology; and 3%, oncology. Ninety-four percent of participants reported educational needs for SHC. With regard to taking responsibility for cancer patients' SHC, 94% of participants responded “Yes.” Only 6% of nurses and 13.3% of physicians had received education concerning SHC.

### SHC Attitudes and Practices According to Demographic Characteristics

Participants' mean scores on the SHCS–A according to demographic characteristics are presented in Table 1. The female and unmarried group significantly encountered barriers in the provision of SHC and had a significantly more negative attitude toward SHC. Participants aged more than 40 years had a significantly more positive attitude when compared to those aged <30 years. There was a significant difference in the level of nurses' and physicians' attitudes regarding SHC.

**Table 1:** Sexual health care attitude and practice according to general characteristics (*N* = 100).

**Category**	***n* (%)**	**SHCS-A**			**SHCS-P**		
		**Mean (*SD*)**	***t* or *F* (P)**		**Mean (*SD*)**	***t* or *F* (P)**	
**Gender**							
Male	23 (26.0)	36.30 (6.66)	3.58 (0.001)	2.75 9.58	4.41 (4.03)	2.207 (0.030)	0.18 3.54
Female	74 (74.0)	30.13 (7.82)			2.55 (3.36)		
**Marriage Status**							
Unmarried	47 (47.0)	29.34 (7.72)	−2.93 (0.004)	−7.58 −1.46	2.26 (3.12)	−1.91 (0.059)	−2.90 0.08
Married	53 (53.0)	33.86 (7.67)			3.68 (3.90)		
**Age**							
<30^a^	38 (38.0)	29.58 (7.96)	3.72 (0.028)		2.00 (2.68)	2.354 (0.101)	
30–39^b^	34 (34.0)	31.58 (8.20)	a < c		3.54 (3.92)		
≥ 40^c^	28 (28.0)	34.85 (6.93)			3.80 (4.11)		
**Religion**			*r* = 0.25 (0.009)			*r* = 0.23 (0.023)	
Yes	48 (48.0)	31.56 (8.54)	−0.21 (0.832)	−3.52 2.84	3.25 (3.86)	0.52 (0.601)	−1.11 1.92
No	52 (52.0)	30.90 (7.51)			2.85 (3.42)		
**Profession**							
Nurse	70 (70.0)	30.17 (7.97)	−3.13 (0.002)	−8.054 −1.91	0.12 (0.16)	−1.69 (0.094)	−3.00 0.23
Physician	30 (30.0)	35.40 (6.82)			0.19 (0.18)		

In terms of SHC practices, male participants provided SHC more frequently than did female participants. Marital status and age group were not associated with SHC practices in the *t*-test or ANOVA. However, a correlation analysis showed that older participants reported a higher frequency of practicing SHC.

### Comparisons of Nurses' and Physicians' SHC Attitudes and Practices

Differences in nurses' and physicians' attitudes and practices regarding SHC in the sum of each domain are shown in Table 2. Compared to nurses, physicians were found to hold a significantly more positive attitude toward SHC, particularly in the domains of discomfort with the provision of SHC, being afraid of colleagues, and lack of environmental support. “Discomfort” refers to uneasy feelings and embarrassment when discussing sexual issues with patients. “Being afraid of colleagues” reflects the lack of prioritization of SHC and poor perception of the extent of one's professional role. “Lack of environmental support” refers to challenges in the work environment, such as insufficient time and the lack of appropriate sites to discuss sexual concerns (15).

**Table 2:** Comparisons between nurses and physicians in sexual health care attitudes and practice (*N* = 100).

**Variables**	**Nurses**	**Physician**	***t* (p)**	**CI**	**Nurses**	**Physician**	***F* (p)**
	**Mean (*****SD*****)**				**Adjusted Mean (*****SE*****)**		
Discomfort	12.37 (4.02)	14.13 (3.55)	−2.07 (0.041)	−3.44 −0.07	14.01 (0.76)	10.30 (1.58)	2.83 (0.095)
Uncertain	7.20 (2.39)	7.80 (1.62)	−1.45 (0.150)	−1.55 0.35	7.44 (0.44)	7.23 (0.92)	0.02 (0.871)
Colleagues	6.44 (2.07)	7.66 (1.21)	−3.68 (0.000)	−2.03 −0.41	6.98 (0.36)	6.40 (0.76)	0.29 (0.590)
Support	4.15 (1.51)	5.80 (1.82)	−4.65 (0.000)	−2.34 −0.94	4.27 (0.32)	5.53 (0.67)	1.79 (0.183)
SHCS–A	30.17 (7.97)	35.40 (6.82)	−3.13 (0.002)	−8.054 −1.91	32.71 (1.52)	29.47 (3.16)	0.54 (0.463)
Reproductive	0.12 (0.27)	0.17 (0.36)	−0.775 (0.440)	−0.18 0.08	0.25 (0.06)	−0.09 (0.12)	4.17 (0.044)
Sexual function	0.02 (0.12)	0.05 (0.15)	−0.803 (0.424)	−0.08 0.03	0.06 (0.02)	−0.03 (0.05)	1.43 (0.234)
Psychological	0.29 (0.39)	0.41 (0.41)	−1.30 (0.194)	−0.29 0.06	0.36 (0.08)	0.27 (0.16)	0.14 (0.706)
Social	0.06 (0.17)	0.12 (0.15)	−1.52 (0.131)	−0.13 0.07	0.12 (0.03)	−0.00 (0.07)	1.74 (0.190)
SHCS-P	0.12 (0.16)	0.19 (0.18)	−1.69 (0.094)	−3.00 0.23	0.18 (0.03)	0.04 (0.07)	2.07 (0.154)

*Discomfort: discomfort with providing sexual health care (seven items). Uncertain: feeling uncertain about a patient's acceptance (four items). Colleagues: afraid of colleagues (three items). Support: lack of environmental support (three items). SHCS-A, Sexual Health Care—Attitude. Sexual function: Practice for sexual function (eight items). Psychological: Practice for psychological factors (six items). Social: Practice for social problems and records (four items). Reproductive: Practice for reproductive care (three items). SHCS-P, Sexual Health Care—Practice*.

Although nurses and physicians are similar in that they are healthcare professionals and collaborate in healthcare provision to patients, they had diverse demographic characteristics; nurses are mainly female and younger, whereas most of the physicians are male and married and older than nurses in this study. Further, these demographic characteristics, including age, sex, and marital status, were associated with SHC attitudes or practices scores in our results, as presented in Table 1. We conducted ANCOVA and estimated the adjusted means after controlling for age, sex, and marital status to explore the differences among nurses and physicians.

In the results, there were no significant differences in attitudes found between nurses and physicians after controlling for demographic variables. However, practices pertaining to reproductive care were performed significantly more by nurses than by physicians (Table 2).

### Correlation Between Attitudes and Practices

There were no significant correlations between the SHCS–A and the SHCS–P for nurses (*r* = 0.158, *p* = 0.196) and physicians (*r* = – 0.082, *p* = 0.665). A small correlation was found between scores on the SHCS–A and the SHCS–P among the groups (*r* = 0.208, *p* = 0.048).

### Participants' Educational Needs

The educational needs reported by participants were broadly related to two domains, knowledge and skill in the practice setting. The following four categories emerged: changes in sexual function, safe sex during treatment, reproductive health, and sexual counseling approaches (Table 3). In terms of educational needs, nurses and physicians reported a need for evidence-based assessment of changes in sexual function after diagnosis, intervention, and counseling. Educational needs included narrative responses such as “It would be helpful to know the sexual concerns of cancer patients according to cancer type in advance,” “I wonder about the possibility of coitus for cancer patients who have low White Blood Cell,” “Information regarding sperm banking for pregnancy among young-adult cancer patients may also be necessary,” and “It would be essential for health professionals to receive sexual counseling as part of their continuing education.” Additionally, participants highlighted the importance of supplementary resources regarding sexual counseling approaches, including the patients' case reports and guidelines regarding SHC during cancer treatment.

**Table 3:** Educational needs of participants: classification of narrative responses.

**Domains**	**Categories**	**Summary of narrative responses**
Knowledge	Changes of sexual function	Sexual concerns among the cancer patients Sexual changes according to the cancer types Assessment tools for evaluating the sexual problems among cancer patients
	Safe sex during the treatments	Adverse effects during coitus and sexual behaviors Negative affect on immune system of sexual intercourse
	Reproductive health	Pregnancy and delivery among the young adult cancer patients Hormonal changes in cancer patients
Skill	Sexual counseling approaches	Making the rapport with cancer patients for sexual counseling Sexual counseling and legal issues Communication between health professionals-patient

## Discussion

The objective of this study was to investigate oncology nurses' and physicians' attitudes and practices regarding cancer patients' SHC. Identifying barriers and potential needs for education could contribute toward the development of continuing education programs for health professionals.

In this study, 94% of nurses and physicians agreed that it was their responsibility to discuss SHC with cancer patients. This result is similar to those of existing studies reporting that health professionals agreed on the need for patients' sexual problems to be addressed when receiving health services (15, 22).

In terms of differences between the nurses and physicians, significant differences were found in the two groups' attitudes. However, there were no differences between the groups after controlling for covariates. These results showed that demographic characteristics including age, sex, and marital status could contribute toward healthcare professionals' SHC attitudes and practices. In our study, after controlling for covariates, reproductive care was found to be more frequently performed by nurses than it was by physicians. The role of the physician in cancer care should be extended to the provision of sexual health as part of cancer treatment, recovery, and survival.

In this study, female, unmarried, and younger participants encountered barriers and had negative attitudes toward the provision of SHC. Participants' age and sex were related to their attitudes regarding cancer patients' sexual health (18, 19). A study on oncology nurses found that younger nurses perceived more barriers in the provision of SHC (16). Another study found that age and sex, coupled with the cultural background of patients and nurses, might be related to healthcare professionals' discomfort with SHC (23, 24). Initiation of discussions about sex by younger health professionals could be perceived as impudence or impoliteness as it would embarrass older patients.

Similarly, the marital status of nurses and physicians might be related to such discomfort. Personal beliefs, values, and morals could also contribute to the provision of SHC (25). Korea is traditionally influenced by Confucian culture, and sexuality is still not openly discussed (23). The Confucian culture of authority, coupled with gender and social norms regarding relationships, could contradict healthcare professionals' roles concerning SHC. Inappropriate assumptions regarding sexual issues should be included in the education programs of nurses and physicians.

Additionally, the participating institution in this study has no specific policy regarding the SHC. The institutional policy might also influence nurses' attitudes and practices. For example, the policy for pre-treatment fertility counseling prescribes that both health professionals and cancer patients are to be given the information and opportunity to decide on the preservation of patients' fertility (6). It is also crucial to conduct bioethical reflections regarding fertility preservation technology and decision making (26). In their investigation of attitudes toward SHC, future studies should also consider differences in participating institutions' approaches and policies in this regard.

In terms of SHC practices, male participants in this study provided SHC more frequently than did their female counterparts. Male staff at cardiac clinics felt more comfortable and assumed more responsibility in the provision of sexual counseling for patients with myocardial infarction (27). In addition, female healthcare professionals have been found to have more difficulties regarding the management of identified patient sexual difficulties (15). The current study results could also inform the development of additional sex-specific educational programs and resources. Specifically, a communication workshop on SHC could be useful in decreasing identified barriers to the provision of SHC (24).

The results of this study facilitate an understanding of the need for educational programs on the provision of SHC to cancer patients. Equipping oncology nurses and physicians with the knowledge to extend their roles by managing patients' sexual function, psychological, and social problems and reproductive care would be effective. Additionally, oncology nurses who are mainly women and relatively younger than physicians ought to enhance their skills in communicating with and counseling male cancer patients. Educational programs could integrate effective strategies to help healthcare professionals overcome discomfort related to age, sex, and marital status, and could help healthcare professionals develop effective communication skills.

Most nurses and physicians in this study had educational needs pertaining to SHC. Although the detail on the type of training received by 6% of nurse respondents and 13.3% of physician respondents were not investigated, they had received few opportunities for training in SHC in Korea. The educational needs reported by nurses and physicians, broadly related to useful knowledge in the practice setting, were changes in sexual function, safe sex during treatment, reproductive health, sexual counseling approaches, and other supplementary resources.

Previous studies have reported that an educational program targeting general patients and high school students, respectively, improved medical students' comfort regarding SHC (23, 28). There was no evidence to say that education will change practices or attitudes. However, considering that there was a small significant correlation between comfort with the SHC provisions and practices in our study, educational programs for healthcare professionals should focus on improving comfort regarding the provision of counseling to cancer patients on sexual concerns, so as to modify the former's attitudes. Moreover, programs should include relevant counseling approaches and the specialized SHC needs of oncology patients, including consideration of the impact of surgery, chemotherapy, and radiotherapy on sexual function and fertility. Details of the educational resources imparted by SHC training programs ought to be investigated.

Further educational initiatives that could be developed as a result of the current study include those focusing on oncology-specific SHC, incorporating sexual problems during and after treatment, including sexual dysfunction, fertility, and reproduction, as well as psychological problems across cancer types. Moreover, participants reported educational needs regarding SHC and counseling approaches, thus also necessitating the incorporation of these into SHC. The above suggest that the gap between healthcare professionals' responsibilities, practices, and attitudinal barriers could be filled through additional education programs and helpful resources, including relevant guidelines.

A limitation of this study is the small sample size and its restriction to the Korean healthcare setting. A more extensive observational study is needed to confirm this study's reproducibility in other countries and cultures. Moreover, the responses provided in the self-reported questionnaires might be biased. The use of convenience sampling, which is a non-probability sampling method, might have also resulted in selection bias. Lastly, the psychometric properties of the SHCS–A and the SHCS–P with use on physicians were not determined; further studies ought to investigate the measures' validity for use on physician, so as to enable assessment of attitudes and practices regarding SHC for cancer patients. The study sample might have been a very diverse group with varying levels of experience with oncology patients including years of experience and level of focus within practice, which would have to be controlled for in the study.

Despite these limitations, this was the first study to compare Korean physicians' and nurses' attitudes and practices regarding SHC for cancer patients. Future research on SHC should address the development of appropriate education programs and practical guidelines for SHC.

## Conclusion

The results of this study provide an understanding of nurses' and physicians' attitudes, practices, and educational needs relating to SHC for cancer patients. The gap between healthcare professionals' responsibilities and practices could be filled through additional education programs and helpful resources, including relevant guidelines. The educational needs reported by nurses and physicians, broadly relating to useful knowledge in the practice setting, are changes in sexual function, safe sex during treatment, reproductive health, sexual counseling approaches, and other supplementary resources.

## Data Availability Statement

The raw data supporting the conclusions of this article will be made available by the authors, without undue reservation.

## Ethics Statement

The study was approved by the Ethics Review Board of Dankook University, Republic of Korea (DKU_IRB-2015_51). All participants provided written consent to take part in the study. Informed consent was obtained from all participants.

## Author Contributions

S-HA contributed to the conceptualization, design of the study, interpretation of the data, and drafting of the manuscript. J-HK contributed to the statistical analysis, interpretation of the data, and drafting of the manuscript. All authors read and approved the final manuscript.

## Conflict of Interest

The authors declare that the research was conducted in the absence of any commercial or financial relationships that could be construed as a potential conflict of interest.
